# Analytic Approximate Solutions to the Boundary Layer Flow Equation over a Stretching Wall with Partial Slip at the Boundary

**DOI:** 10.1371/journal.pone.0149334

**Published:** 2016-03-31

**Authors:** Remus-Daniel Ene, Vasile Marinca, Bogdan Marinca

**Affiliations:** 1 Department of Mathematics, University Politehnica Timişoara, Timişoara, Romania; 2 Department of Mechanics and Vibration, University Politehnica Timişoara, Timişoara, Romania; Department of Electromechanics and Vibration, Center for Advanced and Fundamental Technical Research, Romania Academy, Timişoara, 300223, Romania; 3 Department of Applied Electronics, University Politehnica Timişoara, Timişoara, Romania; North China Electric Power University, CHINA

## Abstract

Analytic approximate solutions using Optimal Homotopy Perturbation Method (OHPM) are given for steady boundary layer flow over a nonlinearly stretching wall in presence of partial slip at the boundary. The governing equations are reduced to nonlinear ordinary differential equation by means of similarity transformations. Some examples are considered and the effects of different parameters are shown. OHPM is a very efficient procedure, ensuring a very rapid convergence of the solutions after only two iterations.

## Introduction

There are a lot of works devoted to the study of hydrodynamic flow over a stretching wall. The dynamics of a such fluid is important in some practical applications. Examples are extrusion of plastic sheet, drawing plastic films, paper production, performance of lubricants and so on. After the pioneering study of Sakiadis [1] on the boundary layer flow over a continuous surface with a constant speed, many researchers have investigated different aspects of this subject. Crane [2] analyzed the boundary layer flow caused by the linearly stretching of an elastic flat surface. Gupta and Gupta [3] extended the work of Crane by including the effect of heat and mass transfer when the surface held at constant temperature. Later, Boutros et al. [4] applied Lie-group method for determining symmetry reductions of partial differential equations and then they determined the solution of steady two-dimensional stagnation point flow of an incompressible viscous fluid over a stretching sheet. Mehmood and Ali [5] investigated the incompressible generalized three-dimensional viscous flow with heat transfer analysis in the presence of viscous dissipation, while Mahapatra et al. [6] studied two-dimensional MHD stagnation-point flow of a viscoelastic fluid toward a stretching surface and Ariel [7] reconsidered the steady, laminar two-dimensional flow of an elastico-viscous fluid when there is a velocity slip on the wall. Ishak et al. presented in [8] the mixed convection two-dimensional boundary layer of a micropolar fluid near the stagnation point on stretching sheet. The transformed ordinary differential equations are solved numererically using the Keller-box method. Misra et al. [9] studied the problem of steady MHD flow of a visco-elastic fluid in a parallel plate channel permeated by a uniform transverse magnetic field in a situation where the surface velocity of the channel varies linearly with distance from the origin. Pal [10] analyzed the two-dimensional stagnation-point flow of an optically dense viscous incompressible fluid over a stretching sheet in the presence of buoyancy forces and thermal radiation, using numerical method. Van Gorder and Vajravelu [11] consider the stretching velocity of the sheet as *u* = *c sgn*(*x*) |*x*|^*n*^, −∞ < *x* < ∞ at *y* = 0 and *n* ≥ 1, assuming the non-slip boundary conditions. Mehmood and Ali reported in [12] the heat transfer analysis in generalized three-dimensional channel flow of a viscous fluid over a stretching sheet heated at constant temperature and taking into account energy losses due to viscous dissipation. The heat transfer problem with a convective boundary condition for a viscous and incompressible fluid over a permeable (with mass flux) stretching / shrinking sheet in a quiscent fluid is investigated by Yao et al. in [13]. Fang et al. [14] considered the behavior of the steady boundary layer flow and heat transfer of a viscous and incompressible fluid over a stretching / shrinking sheet. Also exact solutions are presented for the Navier-Stokes equations, including linearly wall problems as well as the asymptotic suction velocity profiles over a moving plate. Munawar et al. [15] studied the effect of squeezing on the rotating flow of electrically conducting viscous fluid in a channel of lower stretching porous wall. The boundary layer flow of a viscous incompressible fluid toward a porous nonlinearly stretching sheet is considered by Mukhopadhyay in [16]. Butt and Ali examined in [17] the entropy effects due to the flow of a viscous fluid in a rotating channel having a lower porous wall which is stretching in its own plane and upper wall squeezing downwards. An incompressible MHD fluid of two-dimensional upper-convected Maxwell fluid over a porous stretching plate is investigated in [18] and [19].

The purpose of the present paper is to study the boundary layer flow of a viscous incompressible fluid over a porous nonlinearly stretching wall. By means of the similarity transformations we convert the partial differential equations for the momentum equation into a boundary value problem of nonlinear ordinary differential equation. In the boundary conditions, we take into consideration the partial slip. To solve the equation of the motion, we introduce a new homotopy approach called the optimal homotopy perturbation method, that produces approximate analytic solutions to nonlinear ordinary differential equation and sometime even the exact solutions. Using the auxiliary functions, whose parameters *C*_1_, *C*_2_, … ensure rapid convergence of the solutions, OHPM provides a simple but rigorous way of controlling and adjusting the convergence of the solutions by optimally determining the parameters *C*_*i*_.

## Formulation of the problem

Consider the steady, two-dimensional flow of a viscous and incompressible fluid past a flat sheet in the plane *y* = 0 of a Cartesian coordinates system. The flow is confined to *y* > 0. Two equal and positive forces are introduced along *x*-axis so that the wall is stretched keeping the origin fixed. If *u* and *v* are the velocity components along *x*- and *y*-directions, then the continuity and momentum equations governing this type of flow, can be written in the form [11, 16]

$$\begin{array}{c} \frac{\partial u}{\partial x}+\frac{\partial v}{\partial y}=0 \end{array}$$ (1)

$$\begin{array}{c} \begin{array}{c} u\frac{\partial u}{\partial x}+v\frac{\partial u}{\partial y}=\nu \frac{\partial^{2}u}{\partial y^{2}} \end{array} \end{array}$$ (2)

The boundary conditions for the velocity components are

$$\begin{array}{c} u={c\;sgn\left(x\right)\;|x|}^{m}+N_{0}{\left|x\right|}^{-\frac{m-1}{2}}\nu \frac{\partial u}{\partial y},\;-\infty <x<\infty \;\;\text{at}\;\;y=0 \end{array}$$ (3)

$$\begin{array}{c} v=V\left(x\right)=-V_{0}\;sgn\left(x\right)\;{\left|x\right|}^{\frac{m-1}{2}}\;\;\text{at}\;\;y=0 \end{array}$$ (4)

$$\begin{array}{c} u(\infty )=0 \end{array}$$ (5)

Defining the similarity variables as

$$\begin{array}{c} \begin{array}{c} \eta ={\left[\frac{c(m+1)}{2\nu }\right]}^{1/2}y\;{\left|x\right|}^{\frac{m-1}{2}}\\ u={c\;sgn\left(x\right)\;|x|}^{m}\;f^{\prime }\left(\eta \right)\\ v=-{\left[\frac{c(m+1)}{2\nu }\right]}^{1/2}\;sgn\left(x\right)\;{\left|x\right|}^{\frac{m-1}{2}}\;\left[f\left(\eta \right)+\frac{m-1}{m+1}\eta f^{\prime }\left(\eta \right)\right] \end{array} \end{array}$$ (6)

where the prime denotes differentiation with respect to *η*, and then substituting Eq (6) into Eqs (2)–(5), the governing equation and the boundary conditions reduced to

$$\begin{array}{c} f^{\prime \prime \prime }+ff^{\prime \prime }-\frac{2m}{m+1}{f^{\prime }}^{2}=0 \end{array}$$ (7)

$$\begin{array}{c} f\left(0\right)=S,\;\;f^{\prime }\left(0\right)=1+\lambda f^{\prime \prime }\left(0\right),\;\;f^{\prime }\left(\infty \right)=0\;. \end{array}$$ (8)

The physical symbols can be found in the Table 1.

**Table 1:** Nomenclature list.

*u*, *v*	are the velocity components along *x*- and *y*-directions, respectively
*ν*	kinematic viscosity: $\nu =\frac{\mu }{\rho }$
*μ*	dynamic viscosity
*ρ*	fluid density
*S*	suction parameter if *S* > 0 and blowing parameter if *S* < 0 $S=V_{0}{\left[\frac{c\nu (m+1)}{2}\right]}^{-1/2}$
*V*(*x*)	velocity of suction if *V*(*x*)>0 and velocity of blowing if *V*(*x*)<0
*m* > 0	nonlinear stretching parameter
*N*_0_	initial value of velocity slip factor
*c* > 0	is a constant
*V*_0_	initial velocity of suction (*V*_0_ > 0) or initial velocity of blowing (*V*_0_ < 0)
λ	slip parameter: $\lambda =N_{0}{\left[\frac{c\nu (m+1)}{2}\right]}^{1/2}$

## Basic ideas of optimal homotopy perturbation method

To explain the basic ideas of the OHPM, for solving nonlinear differential equation, we consider the following general equation:

$$\begin{array}{c} L\left[f,f^{\prime },f^{\prime \prime },f^{\prime \prime \prime }\right]+N\left[f,f^{\prime },f^{\prime \prime },f^{\prime \prime \prime }\right]=0,\;\eta \in \Omega \end{array}$$ (9)

where *L* is a linear operator and *N* is a nonlinear operator, subject to the boundary / initial conditions:

$$\begin{array}{c} B\left(f,\frac{df}{d\eta }\right)=0,\;\eta \in \Gamma \end{array}$$ (10)

Γ being the boundary of the domain Ω.

We construct a homotopy H(η,p):Ω×[0,1]→IR which satisfies [20]

$$\begin{array}{c} H\left(\eta ,p\right)=L\left(f,f^{\prime },f^{\prime \prime },f^{\prime \prime \prime }\right)+pN\left(f,f^{\prime },f^{\prime \prime },f^{\prime \prime \prime }\right)=0 \end{array}$$ (11)

where *p* ∈ [0, 1] is an embedding parameter. Since Eq (11) implies that

$$\begin{array}{c} H\left(\eta ,0\right)=L\left(f,f^{\prime },f^{\prime \prime },f^{\prime \prime \prime }\right)=0 \end{array}$$ (12)

$$\begin{array}{c} H\left(\eta ,1\right)=L\left(f,f^{\prime },f^{\prime \prime },f^{\prime \prime \prime }\right)+N\left(f,f^{\prime },f^{\prime \prime },f^{\prime \prime \prime }\right)=0 \end{array}$$ (13)

the changing of *p* from zero to unity corresponds to the changing of H(η,p) from *f*_0_(*η*) to *f*(*η*), where *f*_0_(*η*) is obtained from Eq (12):

$$\begin{array}{c} L\left(f_{0},f_{0}^{\prime },f_{0}^{\prime \prime },f_{0}^{\prime \prime \prime }\right)=0 \end{array}$$ (14)

If we assume that the solutions of Eqs (9) and (10) can be expressed as

$$\begin{array}{c} \bar{f}\left(\eta ,p\right)=f_{0}\left(\eta \right)+pf_{1}\left(\eta \right)+p^{2}f_{2}\left(\eta \right) \end{array}$$ (15)

then the approximate solutions of Eqs (9) and (10) can be readily obtained by taking *p* = 1 into Eq (15):

$$\begin{array}{c} \bar{f}\left(\eta \right)=f_{0}\left(\eta \right)+f_{1}\left(\eta \right)+f_{2}\left(\eta \right). \end{array}$$ (16)

Now, applying the Taylor series theorem for *N* and real *α*, *β*, *γ*, we obtain

$$\begin{array}{c} \begin{array}{c} N\left(f,f^{\prime }+\alpha ,f^{\prime \prime }+\beta ,f^{\prime \prime \prime }+\gamma \right)=N\left(f,f^{\prime },f^{\prime \prime },f^{\prime \prime \prime }\right)+\frac{\alpha }{1!}N_{f}\left(f,f^{\prime },f^{\prime \prime },f^{\prime \prime \prime }\right)+\\ +\frac{\beta }{1!}N_{f^{\prime }}\left(f,f^{\prime },f^{\prime \prime },f^{\prime \prime \prime }\right)+\frac{\gamma }{1!}N_{f^{\prime \prime }}\left(f,f^{\prime },f^{\prime \prime },f^{\prime \prime \prime }\right)+... \end{array} \end{array}$$ (17)

where $N_{f}=\frac{\partial N}{\partial f}$. We introduce a number of unknown auxiliary functions *K*_11_, *K*_21_, *K*_22_, *K*_23_ and *K*_24_ depending on the independent variable *η* and some parameters *C*_1_, *C*_2_, … , *C*_*s*_ and a new homotopy which satisfies the following equation [21], [22], [23]:

$$\begin{array}{c} \begin{array}{c} H\left(\eta ,p\right)=L\left(\bar{f},{\bar{f}}^{\prime },{\bar{f}}^{\prime \prime },{\bar{f}}^{\prime \prime \prime }\right)+pK_{11}\left(\eta ,C_{k}\right)N\left(f_{0},f_{0}^{\prime },f_{0}^{\prime \prime },f_{0}^{\prime \prime \prime }\right)+\\ +p^{2}\left[K_{21}\left(\eta ,C_{k}\right)f_{1}N_{f}\left(f_{0},f_{0}^{\prime },f_{0}^{\prime \prime },f_{0}^{\prime \prime \prime }\right)+K_{22}\left(\eta ,C_{k}\right)f_{1}^{\prime }N_{f^{\prime }}\left(f_{0},f_{0}^{\prime },f_{0}^{\prime \prime },f_{0}^{\prime \prime \prime }\right)+\right.\\ \left.+K_{23}\left(\eta ,C_{k}\right)f_{1}^{\prime \prime }N_{f^{\prime \prime }}\left(f_{0},f_{0}^{\prime },f_{0}^{\prime \prime },f_{0}^{\prime \prime \prime }\right)+K_{24}\left(\eta ,C_{k}\right)f_{1}^{\prime \prime \prime }N_{f^{\prime \prime \prime }}\left(f_{0},f_{0}^{\prime },f_{0}^{\prime \prime },f_{0}^{\prime \prime \prime }\right)\right]+...=0 \end{array} \end{array}$$ (18)

where $\bar{f}$ is given by Eq (15) and *k* = 1, 2, …. Equating the coefficients of like powers of *p* into Eq (18), we obtain the following linear equations:

$$\begin{array}{c} L\left(f_{0},f_{0}^{\prime },f_{0}^{\prime \prime },f_{0}^{\prime \prime \prime }\right)=0,\;B\left(f_{0},\;f_{0}^{\prime }\right)=0 \end{array}$$ (19)

$$\begin{array}{c} L\left(f_{1},f_{1}^{\prime },f_{1}^{\prime \prime },f_{1}^{\prime \prime \prime }\right)+K_{11}\left(\eta ,C_{k}\right)N\left(f_{0},f_{0}^{\prime },f_{0}^{\prime \prime },f_{0}^{\prime \prime \prime }\right)=0,\;B\left(f_{1},\;f_{1}^{\prime }\right)=0 \end{array}$$ (20)

$$\begin{array}{c} \begin{array}{c} L\left(f_{2},f_{2}^{\prime },f_{2}^{\prime \prime },f_{2}^{\prime \prime \prime }\right)+K_{21}\left(\eta ,C_{k}\right)f_{1}N_{f}\left(f_{0},f_{0}^{\prime },f_{0}^{\prime \prime },f_{0}^{\prime \prime \prime }\right)+\\ +K_{22}\left(\eta ,C_{k}\right)f_{1}^{\prime }N_{f^{\prime }}\left(f_{0},f_{0}^{\prime },f_{0}^{\prime \prime },f_{0}^{\prime \prime \prime }\right)+K_{23}\left(\eta ,C_{k}\right)f_{1}^{\prime \prime }N_{f^{\prime \prime }}\left(f_{0},f_{0}^{\prime },f_{0}^{\prime \prime },f_{0}^{\prime \prime \prime }\right)+\\ +K_{24}\left(\eta ,C_{k}\right)f_{1}^{\prime \prime \prime }N_{f^{\prime \prime \prime }}\left(f_{0},f_{0}^{\prime },f_{0}^{\prime \prime },f_{0}^{\prime \prime \prime }\right)=0,\;B\left(f_{2},\;f_{2}^{\prime }\right)=0 \end{array} \end{array}$$ (21)

The auxiliary functions *K*_*ij*_ from Eqs (20) and (21) are not unique and they can be chosen so that the products *K*_*ij*_ ⋅ *N*_*a*_ and *N*_*a*_ be of the same form. The convergence-control parameters *C*_*k*_, *k* = 1, 2, … which appear in the expression of the auxiliary functions *K*_*ij*_(*η*, *C*_*k*_) can be optimally determined. This can be done via various methods such as the least-squares method, the weighted residual, the collocation method, the Galerkin method and so on.

In this way, in general, we expect that only two iterations are needed to achieve accurate solutions using OHPM.

It is clear that OHPM is an extension of homotopy perturbation method (HPM), but our procedure is based upon a new construction of the homotopy given by Eq (18), and also it should be emphasize that the presence of the auxiliary functions *K*_*ij*_ which depend of a number of convergence-control parameters *C*_*i*_. These parameters assure the convergence of the solutions. There are examples for which HPM series fail to converge to the true solution. In [24], Turkyilmazoglu resolved the question of convergence of HPM and the internal of convergence series is established. A comparison between HPM and an another analytic method, namely homotopy analysis method (HAM) is given in [25]. In a series of papers [26], [27], [28], [29], [30], the homotopy technique which involves a convergence-control by means of an accelerator parameter is employed to obtain explicitly expressions for different types of nonlinear differential equations.

Approximate analytic solutions are obtained by means of HPM in combination with differential transformation method (DTM) and the Padé approximants in [31] and [32]. Unlike of other methods for example variational iteration method [33], Adomian decomposition method [34], DTM and HPM, optimal homotopy perturbation method lead to a very accurate solution and rapidly converging to the exact solution, using only two iterations. OHPM is an original concept: instead of an infinite series, we need only a few terms in the composition of the approximate solutions. The cornerstone of our technique is its fast convergence via auxiliary functions with a simple but rigorous way, which prove that the optimal method is very efficient in the practical examples. We remark that the linear operator *L* is not unique and can be chosen such that Eq (19) to be verified.

## Application of OHPM to the boundary layer equation over a stretching wall with partial slip at the boundary

In what follows we use the basic ideas of the OHPM, choosing the linear operator in the form:

$$\begin{array}{c} L\left(f,f^{\prime },f^{\prime \prime },f^{\prime \prime \prime }\right)=f^{\prime \prime \prime }+Kf^{\prime \prime }. \end{array}$$ (22)

In this case, Eq (19) becomes

$$\begin{array}{c} \begin{array}{c} f_{0}^{\prime \prime \prime }+Kf_{0}^{\prime \prime }=0,\;\;f_{0}\left(0\right)=S,\;\;f_{0}^{\prime }\left(0\right)=1+\lambda f_{0}^{\prime \prime }\left(0\right),\;\;f_{0}^{\prime }\left(\infty \right)=0 \end{array} \end{array}$$ (23)

which has the following solution

$$\begin{array}{c} f_{0}\left(\eta \right)=S+\frac{1-e^{-K\eta }}{K(1+\lambda K)} \end{array}$$ (24)

where *K* > 0 is an unknown parameter in this moment.

But the linear operator *L* which is not unique, can be chosen as

$$\begin{array}{c} \begin{array}{c} L\left(f,f^{\prime },f^{\prime \prime },f^{\prime \prime \prime }\right)=f^{\prime \prime \prime }-K^{2}f^{\prime } \end{array} \end{array}$$ (25)

and so on. In this work we use the linear operator given by Eq (22), such that the nonlinear operator is obtained from Eqs (22) and (7):

$$\begin{array}{c} \begin{array}{c} N\left(f,f^{\prime },f^{\prime \prime }\right)=\left(f-K\right)f^{\prime \prime }-\frac{2m}{m+1}{f^{\prime }}^{2} \end{array}. \end{array}$$ (26)

From Eq (26) one can get

$$\begin{array}{c} \begin{array}{c} N_{f}\left(f,f^{\prime },f^{\prime \prime }\right)=f^{\prime \prime },\;\;N_{f^{\prime }}\left(f,f^{\prime },f^{\prime \prime }\right)=-\frac{4m}{m+1}f^{\prime },\\ N_{f^{\prime \prime }}\left(f,f^{\prime },f^{\prime \prime }\right)=\left(f-K\right) \end{array}. \end{array}$$ (27)

Substituting Eq (24) into Eqs (26) and (27), one gets

$$\begin{array}{c} \begin{array}{c} N\left(f_{0},f_{0}^{\prime },f_{0}^{\prime \prime }\right)=\frac{\left(1+\lambda K\right)\left(K^{2}-KS\right)-1}{{\left(1+\lambda K\right)}^{2}}e^{-K\eta }+\frac{1-m}{\left(1+m\right){\left(1+\lambda K\right)}^{2}}e^{-2K\eta } \end{array} \end{array}$$ (28)

$$\begin{array}{c} \begin{array}{c} N_{f}\left(f_{0},f_{0}^{\prime },f_{0}^{\prime \prime }\right)=\frac{-K}{1+\lambda K}e^{-K\eta },\;\;N_{f^{\prime }}\left(f_{0},f_{0}^{\prime },f_{0}^{\prime \prime }\right)=-\frac{4m}{\left(1+m)(1+\lambda K\right)}e^{-K\eta },\\ N_{f^{\prime \prime }}\left(f_{0},f_{0}^{\prime },f_{0}^{\prime \prime }\right)=S-K+\frac{1-e^{K\eta }}{K(1+\lambda K)} \end{array}. \end{array}$$ (29)

It should be emphasized that if $N(f_{0},f_{0}^{\prime },f_{0}^{\prime \prime },f_{0}^{\prime \prime \prime })=0$ then the Eqs (7) and (8) are exactly solvable. This special case is *m* = 1 and λ*K*^3^ + (1 − λ*S*)*K*^2^ − *SK* − 1 = 0. The last equation has unique solution if $D=\frac{{\left(2+3\lambda S-3\lambda^{2}S^{2}-2\lambda^{3}S^{3}-27\lambda^{2}\right)}^{2}-4{\left(1+\lambda S+\lambda^{2}S^{2}\right)}^{3}}{54\lambda^{6}}>0$ and three solutions if *D* ≤ 0.

On the other hand, if $\frac{2m}{m+1}=-1$ (or $m=-\frac{1}{3}$), Eqs (7) and (8) become:

$$\begin{array}{c} \begin{array}{c} f^{\prime \prime \prime }+ff^{\prime \prime }+{f^{\prime }}^{2}=0,\;\;\;f\left(0\right)=S,\;\;f^{\prime }\left(0\right)=1+\lambda f^{\prime \prime }\left(0\right),\;\;f^{\prime }\left(\infty \right)=0 \end{array}. \end{array}$$ (30)

By integrating Eq (30) one gets:

$$\begin{array}{c} \begin{array}{c} f^{\prime \prime }\left(\eta \right)+f\left(\eta \right)f^{\prime }\left(\eta \right)=C_{1}=S+\left(1+\lambda S\right)f^{\prime \prime }\left(0\right),\\ f\left(0\right)=S,\;\;f^{\prime }\left(0\right)=1+\lambda f^{\prime \prime }\left(0\right),\;\;f^{\prime }\left(\infty \right)=0. \end{array} \end{array}$$ (31)

Now by integrating Eq (31) once more, it holds that

$$\begin{array}{c} \begin{array}{c} f^{\prime }\left(\eta \right)+\frac{1}{2}f^{2}\left(\eta \right)=\left[S+\left(1+\lambda S\right)f^{\prime \prime }\left(0\right)\right]\eta +C_{2} \end{array}. \end{array}$$ (32)

where the constant *C*_2_ is a given by $C_{2}=f^{\prime }\left(0\right)+\frac{1}{2}f^{2}\left(0\right)$. For *η* → ∞ into Eq (32), it follows that $f^{\prime \prime }\left(0\right)=-\frac{S}{1+\lambda S}$ such that from Eq (8) one can put $f^{\prime }\left(0\right)=\frac{1}{1+\lambda S}$ and therefore $C_{2}=\frac{\lambda S^{3}+S+2}{2(1+\lambda S)}$.

Eq (32) may be written as

$$\begin{array}{c} \begin{array}{c} f^{\prime }\left(\eta \right)+\frac{1}{2}f^{2}\left(\eta \right)=\frac{\lambda S^{3}+S+2}{2(1+\lambda S)} \end{array}. \end{array}$$ (33)

which is a Riccati equation with the solution

$$\begin{array}{c} \begin{array}{c} f\left(\eta \right)={\left(\frac{\lambda S^{3}+S+2}{2(1+\lambda S)}\right)}^{\frac{1}{2}}\tanh\left({\left(\frac{\lambda S^{3}+S+2}{1+\lambda S}\right)}^{\frac{1}{2}}\left(\eta -\eta_{0}\right)\right),\;\;1+\lambda S\neq 0 \end{array} \end{array}$$ (34)

where *η*_0_ is a constant which can be determined from Eq (8):

$$\tanh\eta_{0}=-S{\left(\frac{\lambda S^{3}+S+2}{2(1+\lambda S)}\right)}^{-\frac{1}{2}}.$$

We point out that using OHPM we can obtain exact solutions of Eqs (7) and (8) in particular cases *m* = 1 and $m=-\frac{1}{3}$. For example, in the case *m* = 1, λ = 1, *S* = 1 we obtain *D* > 0 and *K* = 1.754877926. The unique solution can be written in the form

$$\begin{array}{c} f\left(\eta \right)=1.754877926-0.754877926e^{-1.3247175\eta } \end{array}$$ (35a)

In the case *m* = 1, λ = 0.35, *S* = 0.75 we obtain *D* < 0 and three solutions for *K*, but only *K* = 1.28615675 fulfilled condition *K* > 0. The exact solution becomes

$$\begin{array}{c} f\left(\eta \right)=1.365849668-0.615849668e^{-1.28615675\eta } \end{array}$$ (35b)

Now, if $N(f_{0},f_{0}^{\prime },f_{0}^{\prime \prime },f_{0}^{\prime \prime \prime })\neq 0$, Eq (20) becomes

$$\begin{array}{c} \begin{array}{c} f_{1}^{\prime \prime \prime }+Kf_{1}^{\prime \prime }+K_{11}\left(\eta ,C_{k}\right)\left(A_{1}e^{-K\eta }+A_{2}e^{-2K\eta }\right)=0\\ f_{1}\left(0\right)=0,\;\;f_{1}^{\prime }\left(0\right)=\lambda f_{1}^{\prime \prime }\left(0\right),\;\;f_{1}^{\prime }\left(\infty \right)=0 \end{array} \end{array}$$ (36)

where

$$\begin{array}{c} \begin{array}{c} A_{1}=\frac{\lambda K^{3}+\left(1-\lambda S\right)K^{2}-SK-1}{{\left(1+\lambda K\right)}^{2}},\;\;\;A_{2}=\frac{1-m}{\left(1+m\right){\left(1+\lambda K\right)}^{2}} \end{array}. \end{array}$$ (37)

It is natural to choose the auxiliary function *K*_11_(*η*, *C*_*k*_) in the form *K*_11_(*η*, *C*_*k*_) = −*C*_1_, where *C*_1_ is an unknown parameter.

The solution of Eq (36) is given by

$$\begin{array}{c} \begin{array}{c} f_{1}\left(\eta ,C_{1}\right)=A_{3}+\left(\frac{A_{1}C_{1}}{K^{2}}\eta +A_{4}\right)e^{-K\eta }-\frac{A_{2}C_{1}}{4K^{3}}e^{-2K\eta } \end{array} \end{array}$$ (38)

where

$$\begin{array}{c} \begin{array}{c} A_{3}=-\frac{4\left(1+2\lambda K\right)A_{1}C_{1}+\left(1+3\lambda K\right)A_{2}C_{1}}{4K^{3}\left(1+\lambda K\right)},\;\;\;A_{4}=\left(A_{1}+\frac{1}{2}A_{2}\right)\frac{\left(1+2\lambda K\right)C_{1}}{K^{3}\left(1+\lambda K\right)} \end{array}. \end{array}$$ (39)

Into Eq (21) we have freedom to choose *K*_21_ = *K*_23_ = *K*_24_ = 0 and

$$\begin{array}{c} \begin{array}{c} K_{22}\left(\eta ,C_{k}\right)=-\frac{\left(m+1\right)K^{2}}{4m}\left[\left(C_{2}\eta +C_{3}\right)e^{K\eta }+C_{4}\eta +C_{5}\right] \end{array} \end{array}$$ (40)

such that Eq (21) can be written as

$$\begin{array}{c} \begin{array}{c} f_{2}^{\prime \prime \prime }+Kf_{2}^{\prime \prime }=\left[\left(C_{2}\eta +C_{3}\right)e^{K\eta }+C_{4}\eta +C_{5}\right]\left[(KA_{1}C_{1}\eta +K^{3}A_{4}-\right.\\ \left.-A_{1}C_{1})e^{-2K\eta }-A_{2}C_{1}e^{-3K\eta }\right],\;\;f_{2}\left(0\right)=0,\;f_{2}^{\prime }\left(0\right)=\lambda f_{2}^{\prime \prime }\left(0\right),\;f_{2}^{\prime }\left(\infty \right)=0 \end{array}. \end{array}$$ (41)

From Eq (41) follows that

$$\begin{array}{c} \begin{array}{c} f_{2}\left(\eta \right)=A_{5}+\left(\frac{A_{1}C_{1}C_{2}}{3K}\eta^{3}+\frac{3A_{1}C_{1}C_{2}+KA_{1}C_{1}C_{3}+K^{3}A_{4}C_{2}}{2K^{2}}\eta^{2}+\right.\\ \left.+\frac{5A_{1}C_{1}C_{2}+A_{1}C_{1}C_{3}+K^{3}A_{4}C_{2}+K^{4}A_{4}C_{3}}{K^{3}}\eta +A_{6}\right)e^{-K\eta }-\left(\frac{A_{1}C_{1}C_{4}}{4K^{2}}\eta^{2}+\right.\\ \left.+\frac{3A_{1}C_{1}C_{4}+KA_{1}C_{1}C_{5}+K^{3}A_{4}C_{4}-A_{2}C_{1}C_{2}}{4K^{3}}\eta +\right.\\ \left.+\frac{3A_{1}C_{1}C_{4}+2KA_{1}C_{1}C_{5}+4K^{3}A_{4}C_{4}-4A_{2}C_{1}C_{2}-2KA_{2}C_{1}C_{3}+2K^{4}A_{4}C_{5}}{8K^{4}}\right)e^{-2K\eta }+ \end{array}\\ \begin{array}{c} +\left(\frac{A_{2}C_{1}C_{4}}{4K^{3}}\eta +\frac{7A_{2}C_{1}C_{4}+6KA_{2}C_{1}C_{5}}{108K^{4}}\right)e^{-3K\eta } \end{array} \end{array}$$ (42)

where

$$\begin{array}{c} A_{5}=\frac{1}{K\left(1+\lambda K\right)}[\left(\frac{5\lambda K-2\lambda +5}{K^{2}}A_{1}+\frac{10\lambda K+5}{4K^{3}}A_{2}\right)C_{1}C_{2}+\\ +\left(\frac{\lambda K-\lambda +1}{K}A_{1}-\frac{3\lambda }{4K}A_{2}\right)C_{1}C_{3}+\left(\frac{5\lambda K-3}{8K^{3}}A_{1}-\frac{106\lambda K+13}{108K^{3}}A_{2}\right)C_{1}C_{4}+\\ +\left(\frac{4\lambda K+1}{9K^{2}}A_{2}-\frac{\lambda K+2}{4K^{2}}A_{1}\right)C_{1}C_{5}+K\left(\lambda K+1\right)A_{4}C_{2}+K^{2}\left(\lambda K+1\right)A_{4}C_{3}-\\ -\frac{6\lambda K+5}{4}A_{4}C_{4}+\frac{\left(3\lambda K+1\right)K}{4}A_{4}C_{5}] \end{array}$$

$$\begin{array}{c} A_{6}=\frac{1}{K\left(1+\lambda K\right)}[-\left(\frac{5\lambda K-2\lambda +5}{K^{2}}A_{1}+\frac{8\lambda K+3}{4K^{3}}A_{2}\right)C_{1}C_{2}+\\ +\left(\frac{2\lambda K-1}{2K^{2}}A_{2}-\frac{\lambda K-\lambda +1}{K}A_{1}\right)C_{1}C_{3}+\left(\frac{33\lambda K+2}{36K^{3}}A_{2}-\frac{\lambda }{K}A_{1}\right)C_{1}C_{4}+\\ +\left(\frac{1}{4K^{2}}A_{1}-\frac{3\lambda K+1}{6K^{2}}A_{2}\right)C_{1}C_{5}-K\left(\lambda K+1\right)A_{4}C_{2}-K^{2}\left(\lambda K+1\right)A_{4}C_{3}+\\ +\left(\frac{3}{4}+\lambda K\right)A_{4}C_{4}-\frac{K}{2}\left(1+2\lambda K\right)A_{4}C_{5}]. \end{array}$$

The second-order approximate solution Eq (16) for Eqs (7) and (8) is obtained from Eqs (24), (38) and (42):

$$\begin{array}{c} \bar{f}\left(\eta ,C_{k}\right)=f_{0}\left(\eta \right)+f_{1}\left(\eta ,C_{1}\right)+f_{2}\left(\eta ,C_{2},C_{3},C_{4},C_{5}\right). \end{array}$$ (43)

**Remark 1.** The choice of the auxiliary functions *K*_*ij*_(*ηC*_*k*_) is not unique. We can choose, for example *K*_11_ = *C*_1_+*C*_2_ *e*^−*Kη*^ or *K*_11_ = *C*_1_+*C*_2_ *e*^−*Kη*^+*C*_3_ *ηe*^−*Kη*^, $K_{22}\left(\eta ,C_{k}\right)=-\frac{\left(m+1\right)K^{2}}{4m}[C_{2}\eta +C_{3}e^{k\eta }+C_{4}\eta +C_{5}+C_{6}e^{-k\eta }]$ or $K_{22}\left(\eta ,C_{k}\right)=-\frac{\left(m+1\right)K^{2}}{4m}[C_{2}\eta +C_{3}e^{k\eta }+C_{4}\eta +C_{5}+\left(C_{6}+C_{7}\eta \right)e^{-k\eta }]$ and so on.

## Numerical examples

In order to prove the accuracy of the obtained results, we will determine the convergence-control parameters *K* and *C*_*i*_ which appear in Eq (43) by means of the least-squares method. In this way, the convergence-control parameters are optimally determined and the second-order approximate solutions are known for different values of the known parameters *m*, λ and *S*. We illustrate the accuracy of the OHPM comparing our approximate solutions with the numerical integration results computed by means of the fourth-order Runge-Kutta method using Wolfram Mathematica 6.0 software in combination with the shooting method. For some values of the parameters *m*, λ and *S* we will determine the approximate solutions.

**Example 5.1** We consider *S* = 0.5, *m* = 1 and λ = 0.3. Following the procedure described above are obtained the convergence-control parameters:

$$C_{1}=-0.6830983363,\;C_{2}=10.7987841221,\;C_{3}=-63.8080306450,$$

$$C_{4}=396.5194058302,\;C_{5}=-350.3111093217,\;K=1.1479603826$$

and consequently, the second-order approximate solution Eq (43) can be written in the form:

$$\begin{array}{c} \begin{array}{c} \bar{f}\left(\eta \right)=1.1479603670+(-0.6479641342+9.9746399237\cdot 10^{-7}\eta +\\ +1.6180226816\cdot 10^{-7}\eta^{2}-4.9003691794\cdot 10^{-8}\eta^{3})e^{-1.1479603826\eta }-\\ -(-3.7672401115\cdot 10^{-6}-3.3199896493\cdot 10^{-6}\eta -\\ -1.1755815194\cdot 10^{-6}\eta^{2})e^{-2.2959207653\eta } \end{array} \end{array}$$ (44)

**Example 5.2** If *S* = 1, *m* = 1 and λ = 0.3, then it hold that

$$C_{1}=-0.0320329745,\;C_{2}=-0.1936363799,\;C_{3}=3.8482361163,$$

$$C_{4}=25.7814460709,\;C_{5}=-1.7865584311,\;K=1.4714959279,\;$$

and therefore the second-order approximate solution Eq (43) is given by

$$\begin{array}{c} \begin{array}{c} \bar{f}\left(\eta \right)=1.4714682042+(-0.4714808668-5.3720872022\cdot 10^{-6}\eta -\\ -1.4339617409\cdot 10^{-6}\eta^{2}+5.6409419274\cdot 10^{-8}\eta^{3})e^{-1.4714959279\eta }-\\ -(-0.0000126626-0.0000109372\eta -\\ -3.8280195392\cdot 10^{-6}\eta^{2})e^{-2.9429918558\eta } \end{array} \end{array}$$ (45)

**Example 5.3** For *S* = 2, *m* = 1 and λ = 0.3 we obtain:

$$C_{1}=-0.7234916519,\;C_{2}=-12.6062261413,\;C_{3}=36.7837409836,$$

$$C_{4}=-462.5125528241,\;C_{5}=234.5213384929,\;K=2.2631742890$$

and

$$\begin{array}{c} \begin{array}{c} \bar{f}\left(\eta \right)=2.2631743862+(-0.2631764693+1.1582907757\cdot 10^{-6}\eta +\\ +2.9862842730\cdot 10^{-7}\eta^{2}-2.0486469642\cdot 10^{-7}\eta^{3})e^{-2.2631742890\eta }-\\ -(-2.0830097026\cdot 10^{-6}-3.5844742210\cdot 10^{-6}\eta -\\ -2.4908571285\cdot 10^{-6}\eta^{2})e^{-4.5263485780\eta } \end{array} \end{array}$$ (46)

**Example 5.4** In the case that *S* = 0.5, *m* = 1 and λ = 0.5 we have

$$C_{1}=-0.0091992214,\;C_{2}=-0.5705954969,\;C_{3}=12.1345916153,$$

$$C_{4}=49.7970575239,\;C_{5}=-1.4069400905,\;K=1.0922123670$$

$$\begin{array}{c} \begin{array}{c} \bar{f}\left(\eta \right)=1.0921935857+(-0.5922075046-4.6537981427\cdot 10^{-6}\eta -\\ -9.5516132587\cdot 10^{-7}\eta^{2}+3.6848536763\cdot 10^{-8}\eta^{3})e^{-1.0922123670\eta }-\\ -(-0.0000139189-8.7390276782\cdot 10^{-6}\eta -\\ -2.2082577433\cdot 10^{-6}\eta^{2})e^{-2.1844247340\eta } \end{array} \end{array}$$ (47)

**Example 5.5** For *S* = 1, *m* = 1 and λ = 0.5 the second-order approximate solution can be written as

$$\begin{array}{c} \begin{array}{c} \bar{f}\left(\eta \right)=1.4142135623+(-0.4142231199-4.4566033252\cdot 10^{-6}\eta -\\ -1.0941638864\cdot 10^{-6}\eta^{2}+5.2422526842\cdot 10^{-8}\eta^{3})e^{-1.4142381988\eta }-\\ -(-9.5575971692\cdot 10^{-6}-7.7735269570\cdot 10^{-6}\eta -\\ -2.5417856349\cdot 10^{-6}\eta^{2})e^{-2.8284763977\eta } \end{array} \end{array}$$ (48)

**Example 5.6** If *S* = 2, *m* = 1 and λ = 0.5 we obtain

$$\begin{array}{c} \begin{array}{c} \bar{f}\left(\eta \right)=2.2143197433+(-0.2143218697+1.1817583669\cdot 10^{-6}\eta +\\ +2.6384310526\cdot 10^{-7}\eta^{2}-1.8475999430\cdot 10^{-7}\eta^{3})e^{-2.2143196048\eta }-\\ -(-2.1263322216\cdot 10^{-6}-3.5612662786\cdot 10^{-6}\eta -\\ -2.4250419185\cdot 10^{-6}\eta^{2})e^{-4.4286392097\eta } \end{array} \end{array}$$ (49)

**Example 5.7** We consider *S* = 0.5, *m* = 1 and λ = 0.75 and therefore

$$\begin{array}{c} \begin{array}{c} \bar{f}\left(\eta \right)=1.0401088461+(-0.5401232188-4.8231615759\cdot 10^{-6}\eta -\\ -9.0210933446\cdot 10^{-7}\eta^{2}+3.1565595846\cdot 10^{-8}\eta^{3})e^{-1.0401296683\eta }-\\ -(-0.0000143726-8.5338553710\cdot 10^{-6}\eta -\\ -2.0164249804\cdot 10^{-6}\eta^{2})e^{-2.0802593367\eta } \end{array} \end{array}$$ (50)

**Example 5.8** In the case *S* = 1, *m* = 1 and λ = 0.75, it holds that

$$\begin{array}{c} \begin{array}{c} \bar{f}\left(\eta \right)=1.3628584281+(-0.3628703689-6.1057142445\cdot 10^{-6}\eta -\\ -1.4076332023\cdot 10^{-6}\eta^{2}+8.1738645538\cdot 10^{-8}\eta^{3})e^{-1.3628949097\eta }-\\ -(-0.0000119407-9.1496040607\cdot 10^{-6}\eta -\\ -2.8026904713\cdot 10^{-6}\eta^{2})e^{-2.7257898194\eta } \end{array} \end{array}$$ (51)

**Example 5.9** For *S* = 2, *m* = 1 and λ = 0.75 the result is

$$\begin{array}{c} \begin{array}{c} \bar{f}\left(\eta \right)=2.1747650648+(-0.1747665309+8.3502378913\cdot 10^{-7}\eta +\\ +1.6318305121\cdot 10^{-7}\eta^{2}-1.1874775356\cdot 10^{-7}\eta^{3})e^{-2.1747648717\eta }-\\ -(-1.4661321304\cdot 10^{-6}-2.3946894463\cdot 10^{-6}\eta -\\ -1.5988887235\cdot 10^{-6}\eta^{2})e^{-4.3495297435\eta } \end{array} \end{array}$$ (52)

**Remark 2.** Using HPM with three iterations, for *m* = 1, we obtain

$$\begin{array}{c} \begin{array}{c} {\bar{f}}_{HPM}\left(\eta \right)=f_{0}\left(\eta \right)+f_{1}\left(\eta \right)+f_{2}\left(\eta \right)+f_{3}\left(\eta \right) \end{array} \end{array}$$ (53)

where

$$\begin{array}{c} \begin{array}{c} f_{0}\left(\eta \right)=S+\frac{1-e^{-\eta }}{\left(1+\lambda \right)}\\ f_{1}\left(\eta \right)=A_{1}\left(\eta +A_{2}\right)\cdot e^{-\eta }+A_{3}\\ f_{2}\left(\eta \right)=B_{1}+B_{2}\cdot \left(B_{3}\eta^{2}+B_{4}\eta +B_{5}\right)\cdot e^{-\eta }\\ f_{3}\left(\eta \right)=N_{1}+\left(N_{2}+N_{3}\eta +N_{4}\eta^{2}+N_{5}\eta^{3}\right)\cdot e^{-\eta } \end{array} \end{array}$$ (54)

with

$$A_{1}=\frac{S+S\lambda -\lambda }{{\left(1+\lambda \right)}^{2}},\;\;\;\;A_{2}=\frac{1+2\lambda }{1+\lambda },\;\;\;\;A_{3}=-\frac{\left(1+2\lambda )\cdot (S+S\lambda -\lambda \right)}{{\left(1+\lambda \right)}^{3}}$$

$$B_{1}=\frac{\left(S+S\lambda -\lambda \right)\cdot \left[S\left(3\lambda^{3}+6\lambda^{2}+4\lambda +1\right)-3\lambda^{3}+\lambda^{2}+3\lambda +1\right]}{{\left(\lambda +1\right)}^{5}}$$

$$B_{2}=\frac{S+S\lambda -\lambda }{{\left(\lambda +1\right)}^{3}},\;\;\;\;B_{3}=-\frac{S+S\lambda -\lambda }{2},\;\;\;\;B_{4}=-\left(S+2S\lambda -2\lambda +3-\frac{2}{\lambda +1}\right)$$

$$B_{5}=\frac{S\left(-3\lambda^{3}-6\lambda^{2}-4\lambda -1\right)+3\lambda^{3}-\lambda^{2}-3\lambda -1}{{\left(\lambda +1\right)}^{2}}$$

$$N_{1}=\frac{1}{1+\lambda }\left(M_{1}+2M_{2}+6M_{3}+2\lambda M_{1}+3\lambda M_{2}+8\lambda M_{3}\right),\;\;\;\;N_{2}=-N_{1}$$

$$N_{3}=-M_{1}-2M_{2}-6M_{3},\;\;\;\;N_{4}=-\frac{1}{2}M_{2}-2M_{3},\;\;\;\;N_{5}=\frac{1}{3}M_{3}$$

where *M*_1_, *M*_2_ and *M*_3_ denote

$$M_{1}=-\frac{1}{1+\lambda }\left(A_{1}A_{3}+B_{1}\right)+B_{2}\left(2B_{3}-2B_{4}+B_{5}\right)\cdot \frac{S+S\lambda -\lambda }{1+\lambda }$$

$$M_{2}=B_{2}\left(-4B_{3}+B_{4}\right)\cdot \frac{S+S\lambda -\lambda }{1+\lambda },\;\;\;\;M_{3}=B_{2}B_{3}\cdot \frac{S+S\lambda -\lambda }{1+\lambda }$$

In Tables 2–10 we present a comparison between the second-order approximate velocities obtained from Eqs (44)–(52), with numerical results. In Table 8 we present a comparison between the results obtained from Eqs (50) and (53) and numerical results.

**Table 2:** Comparison between OHPM results for velocity obtained from Eq (44) and numerical results for *S* = 0.5, *m* = 1, λ = 0.3.

*η*	$f_{\text{numerical}}^{\prime }$	${\bar{f}}_{\text{OHPM}}^{\prime }$ from Eq (44)	$\begin{array}{c} \text{relative error}=|f_{\text{numerical}}^{\prime }-{\bar{f}}_{\text{OHPM}}^{\prime }| \end{array}$
0	0.7438328206	0.7438328236	2.9 ⋅ 10^−9^
4/5	0.2969158780	0.2969156191	2.5 ⋅ 10^−7^
8/5	0.1185197890	0.1185197758	1.3 ⋅ 10^−8^
12/5	0.0473095084	0.0473095183	9.8 ⋅ 10^−9^
16/5	0.0188845324	0.0188845270	5.3 ⋅ 10^−9^
4	0.0075381357	0.0075381309	4.8 ⋅ 10^−9^
24/5	0.0030089964	0.0030089942	2.2 ⋅ 10^−9^
28/5	0.0012010990	0.0012011004	1.3 ⋅ 10^−9^
32/5	0.0004794428	0.0004794437	8.9 ⋅ 10^−10^
36/5	0.0001913786	0.0001913800	1.3 ⋅ 10^−9^
8	0.0000763925	0.0000763934	8.6 ⋅ 10^−10^

**Table 3:** Comparison between OHPM results for velocity obtained from Eq (45) and numerical results for *S* = 1, *m* = 1, λ = 0.3.

*η*	$f_{\text{numerical}}^{\prime }$	${\bar{f}}_{\text{OHPM}}^{\prime }$ from Eq (45)	$\begin{array}{c} \text{relative error}=|f_{\text{numerical}}^{\prime }-{\bar{f}}_{\text{OHPM}}^{\prime }| \end{array}$
0	0.6937504717	0.6937504747	2.9 ⋅ 10^−9^
4/5	0.2137779815	0.2137779360	4.5 ⋅ 10^−8^
8/5	0.0658752766	0.0658752913	1.4 ⋅ 10^−8^
12/5	0.0202993432	0.0202993413	1.9 ⋅ 10^−9^
16/5	0.0062552064	0.0062552019	4.5 ⋅ 10^−9^
4	0.0019275303	0.0019275298	5.1 ⋅ 10^−10^
24/5	0.0005939648	0.0005939659	1.09 ⋅ 10^−9^
28/5	0.0001830287	0.0001830301	1.4 ⋅ 10^−9^
32/5	0.0000563999	0.0000564006	7.2 ⋅ 10^−10^
36/5	0.0000173785	0.0000173798	1.3 ⋅ 10^−9^
8	5.35550279 ⋅ 10^−6^	5.35561322 ⋅ 10^−6^	1.104 ⋅ 10^−10^

**Table 4:** Comparison between OHPM results for velocity obtained from Eq (46) and numerical results for *S* = 2, *m* = 1, λ = 0.3.

*η*	$f_{\text{numerical}}^{\prime }$	${\bar{f}}_{\text{OHPM}}^{\prime }$ from Eq (46)	$\begin{array}{c} \text{relative error}=|f_{\text{numerical}}^{\prime }-{\bar{f}}_{\text{OHPM}}^{\prime }| \end{array}$
0	0.5956095301	0.5956095331	2.9 ⋅ 10^−9^
4/5	0.0974213944	0.0974214024	7.9 ⋅ 10^−9^
8/5	0.0159348140	0.0159348111	2.9 ⋅ 10^−9^
12/5	0.0026063923	0.0026063902	2.04 ⋅ 10^−9^
16/5	0.0004263170	0.0004263177	7.1 ⋅ 10^−10^
4	0.0000697308	0.0000697316	8.6 ⋅ 10^−10^
24/5	0.0000114057	0.0000114059	1.6 ⋅ 10^−10^
28/5	1.86567247 ⋅ 10^−6^	1.86567721 ⋅ 10^−6^	4.7 ⋅ 10^−12^
32/5	3.04991333 ⋅ 10^−7^	3.05174513 ⋅ 10^−7^	1.8 ⋅ 10^−10^
36/5	4.93710461 ⋅ 10^−8^	4.99191425 ⋅ 10^−8^	5.4 ⋅ 10^−10^
8	8.58093734 ⋅ 10^−9^	8.16571298 ⋅ 10^−9^	4.1 ⋅ 10^−10^

**Table 5:** Comparison between OHPM results for velocity obtained from Eq (47) and numerical results for *S* = 0.5, *m* = 1, λ = 0.5.

*η*	$f_{\text{numerical}}^{\prime }$	${\bar{f}}_{\text{OHPM}}^{\prime }$ from Eq (47)	$\begin{array}{c} \text{relative error}=|f_{\text{numerical}}^{\prime }-{\bar{f}}_{\text{OHPM}}^{\prime }| \end{array}$
0	0.6467900358	0.6467900408	5.0 ⋅ 10^−9^
4/5	0.2699581329	0.2699581155	1.7 ⋅ 10^−8^
8/5	0.1126755034	0.1126755123	8.8 ⋅ 10^−9^
12/5	0.0470286633	0.0470286605	2.7 ⋅ 10^−9^
16/5	0.0196288865	0.0196288817	4.8 ⋅ 10^−9^
4	0.0081927295	0.0081927273	2.1 ⋅ 10^−9^
24/5	0.0034194941	0.0034194918	2.2 ⋅ 10^−9^
28/5	0.0014272337	0.0014272329	7.5 ⋅ 10^−10^
32/5	0.0005957000	0.0005957011	1.02 ⋅ 10^−9^
36/5	0.0002486342	0.0002486349	7.4 ⋅ 10^−10^
8	0.0001037749	0.0001037758	8.9 ⋅ 10^−10^

**Table 6:** Comparison between OHPM results for velocity obtained from Eq (48) and numerical results for *S* = 1, *m* = 1, λ = 0.5.

*η*	$f_{\text{numerical}}^{\prime }$	${\bar{f}}_{\text{OHPM}}^{\prime }$ from Eq (48)	$\begin{array}{c} \text{relative error}=|f_{\text{numerical}}^{\prime }-{\bar{f}}_{\text{OHPM}}^{\prime }| \end{array}$
0	0.5857864376	0.5857864426	4.9 ⋅ 10^−9^
4/5	0.1889693005	0.1889692757	2.4 ⋅ 10^−8^
8/5	0.0609597509	0.0609597447	6.2 ⋅ 10^−9^
12/5	0.0196650461	0.0196650423	3.7 ⋅ 10^−9^
16/5	0.0063437610	0.0063437580	3.03 ⋅ 10^−9^
4	0.0020464387	0.0020464380	7.09 ⋅ 10^−10^
24/5	0.0006601633	0.0006601627	6.8 ⋅ 10^−10^
28/5	0.0002129620	0.0002129628	7.6 ⋅ 10^−10^
32/5	0.0000686993	0.0000687000	7.3 ⋅ 10^−10^
36/5	0.0000221622	0.0000221620	1.4 ⋅ 10^−10^
8	7.15026166 ⋅ 10^−6^	7.14930719 ⋅ 10^−6^	9.5 ⋅ 10^−10^

**Table 7:** Comparison between OHPM results for velocity obtained from Eq (49) and numerical results for *S* = 2, *m* = 1, λ = 0.5.

*η*	$f_{\text{numerical}}^{\prime }$	${\bar{f}}_{\text{OHPM}}^{\prime }$ from Eq (49)	$\begin{array}{c} \text{relative error}=|f_{\text{numerical}}^{\prime }-{\bar{f}}_{\text{OHPM}}^{\prime }| \end{array}$
0	0.4745724391	0.4745724441	4.9 ⋅ 10^−9^
4/5	0.0807177531	0.0807177601	6.9 ⋅ 10^−9^
8/5	0.0137288924	0.0137288917	6.5 ⋅ 10^−10^
12/5	0.0023350821	0.0023350799	2.1 ⋅ 10^−9^
16/5	0.0003971633	0.0003971636	3.1 ⋅ 10^−10^
4	0.0000675515	0.0000675523	7.4 ⋅ 10^−10^
24/5	0.0000114898	0.0000114898	3.6 ⋅ 10^−11^
28/5	1.95429998 ⋅ 10^−6^	1.95432255 ⋅ 10^−6^	2.2 ⋅ 10^−11^
32/5	3.31926594 ⋅ 10^−7^	3.32417347 ⋅ 10^−7^	4.9 ⋅ 10^−10^
36/5	5.59263376 ⋅ 10^−8^	5.65430171 ⋅ 10^−8^	6.1 ⋅ 10^−10^
8	9.65723477 ⋅ 10^−9^	9.61796719 ⋅ 10^−9^	3.9 ⋅ 10^−11^

**Table 8:** Comparison between OHPM results for velocity obtained from Eq (50) and numerical results for *S* = 0.5, *m* = 1, λ = 0.75 $\left(\varepsilon_{1}=\;|{f^{\prime }}_{\text{numerical}}-{{\bar{f}}^{\prime }}_{\text{HPM}}|\;\varepsilon_{2}=\;|{f^{\prime }}_{\text{numerical}}-{{\bar{f}}^{\prime }}_{\text{OHPM}}|\right)$.

*η*	$f_{\text{numerical}}^{\prime }$	${\bar{f}}_{\text{HPM}}^{\prime }$ from Eq (53)	${\bar{f}}_{\text{OHPM}}^{\prime }$ from Eq (50)	*ε*_1_	*ε*_2_
0	0.5617719887	0.5560723605203368	0.5617719962	5.69 ⋅ 10^−3^	7.5 ⋅ 10^−9^
4/5	0.2444493881	0.2370356141324056	0.2444495518	7.41 ⋅ 10^−3^	1.6 ⋅ 10^−7^
8/5	0.1063698285	0.1007809946068748	0.1063698346	5.58 ⋅ 10^−3^	6.06 ⋅ 10^−9^
12/5	0.0462857799	0.0427175382018216	0.0462857850	3.56 ⋅ 10^−3^	5.04 ⋅ 10^−9^
16/5	0.0201408017	0.0180397255093539	0.0201407971	2.101 ⋅ 10^−3^	4.6 ⋅ 10^−9^
4	0.0087640694	0.0075844395742249	0.0087640663	1.17 ⋅ 10^−3^	3.04 ⋅ 10^−9^
24/5	0.0038135995	0.0031716219952952	0.0038135969	6.41 ⋅ 10^−4^	2.6 ⋅ 10^−9^
28/5	0.0016594507	0.0013176217558175	0.0016594499	3.41 ⋅ 10^−4^	8.4 ⋅ 10^−10^
32/5	0.0007220963	0.0005429862202446	0.0007220940	1.79 ⋅ 10^−4^	2.2 ⋅ 10^−9^
36/5	0.0003142113	0.0002215120273857	0.0003142126	9.26 ⋅ 10^−5^	1.3 ⋅ 10^−9^
8	0.0001367261	0.0000892104089751	0.0001367268	4.75 ⋅ 10^−5^	6.8 ⋅ 10^−10^

**Table 9:** Comparison between OHPM results for velocity obtained from Eq (51) and numerical results for *S* = 1, *m* = 1, λ = 0.75.

*η*	$f_{\text{numerical}}^{\prime }$	${\bar{f}}_{\text{OHPM}}^{\prime }$ from Eq (51)	$\begin{array}{c} \text{relative error}=|f_{\text{numerical}}^{\prime }-{\bar{f}}_{\text{OHPM}}^{\prime }| \end{array}$
0	0.4945246671	0.4945246746	7.5 ⋅ 10^−9^
4/5	0.1662196474	0.1662196801	3.2 ⋅ 10^−8^
8/5	0.0558697909	0.0558697851	5.7 ⋅ 10^−9^
12/5	0.0187789396	0.0187789516	1.1 ⋅ 10^−8^
16/5	0.0063119834	0.0063119803	3.08 ⋅ 10^−9^
4	0.0021215848	0.0021215838	1.02 ⋅ 10^−9^
24/5	0.0007131092	0.0007131077	1.4 ⋅ 10^−9^
28/5	0.0002396912	0.0002396903	9.4 ⋅ 10^−10^
32/5	0.0000805642	0.0000805649	7.1 ⋅ 10^−10^
36/5	0.0000270773	0.0000270795	2.2 ⋅ 10^−9^
8	9.102173001 ⋅ 10^−6^	9.101998883 ⋅ 10^−6^	1.7 ⋅ 10^−10^

**Table 10:** Comparison between OHPM results for velocity obtained from Eq (52) and numerical results for *S* = 2, *m* = 1, λ = 0.75.

*η*	$f_{\text{numerical}}^{\prime }$	${\bar{f}}_{\text{OHPM}}^{\prime }$ from Eq (52)	$\begin{array}{c} \text{relative error}=|f_{\text{numerical}}^{\prime }-{\bar{f}}_{\text{OHPM}}^{\prime }| \end{array}$
0	0.3800729574	0.3800729649	7.4 ⋅ 10^−9^
4/5	0.0667230985	0.0667231040	5.5 ⋅ 10^−9^
8/5	0.0117134659	0.0117134640	1.9 ⋅ 10^−9^
12/5	0.0020563388	0.0020563372	1.6 ⋅ 10^−9^
16/5	0.0003609976	0.0003609977	6.2 ⋅ 10^−11^
4	0.0000633743	0.0000633748	5.3 ⋅ 10^−10^
24/5	0.0000111255	0.0000111258	2.6 ⋅ 10^−10^
28/5	1.95315423 ⋅ 10^−6^	1.95323001 ⋅ 10^−6^	7.5 ⋅ 10^−11^
32/5	3.42589685 ⋅ 10^−7^	3.42909134 ⋅ 10^−7^	3.1 ⋅ 10^−10^
36/5	6.03175349 ⋅ 10^−8^	6.020200121 ⋅ 10^−8^	1.1 ⋅ 10^−10^
8	1.04328341 ⋅ 10^−8^	1.05693898 ⋅ 10^−8^	1.3 ⋅ 10^−10^

In Table 11 we present a comparison between the skin-friction coefficient $-{\bar{f}}^{\prime \prime }\left(0\right)$ obtained by means of OHAM from Eqs (44)–(52) with numerical results and HPM and corresponding relative errors for *m* = 1 and different values of the parameters *S* and λ, while in Table 12 we present a comparison between the entrainment parameter $\bar{f}\left(\infty \right)$ obtained from Eqs (44)–(52) with numerical results and corresponding relative errors.

**Table 11:** Comparison between skin-friction coefficient obtained by numerical method and OHPM for *m* = 1 and different values of the parameters *S* and λ.

		skin-friction coefficient: −*f*′′(0)		
*S*	λ	−*f*′′_numeric_(0)	$-{{\bar{f}}^{\prime \prime }}_{\text{OHPM}}\left(0\right)$	$\begin{array}{c} \text{relative error}=|{f^{\prime \prime }}_{\text{numeric}}\left(0\right)-{{\bar{f}}^{\prime \prime }}_{\text{OHPM}}\left(0\right)| \end{array}$
0.5	0.3	0.8538905977	0.8538905877	9.9 ⋅ 10^−9^
1	0.3	1.0208317608	1.0208317508	1.0 ⋅ 10^−8^
2	0.3	1.3479682328	1.3479682228	9.9 ⋅ 10^−9^
0.5	0.5	0.7064199283	0.7064199183	9.9 ⋅ 10^−9^
1	0.5	0.8284271247	0.8284271147	1.0 ⋅ 10^−8^
2	0.5	1.0508551216	1.0508551116	9.9 ⋅ 10^−9^
0.5	0.75	0.5843040150	0.5843040050	1.0 ⋅ 10^−8^
1	0.75	0.6739671105	0.6739671005	9.9 ⋅ 10^−9^
2	0.75	0.8265693900	0.8265693800	9.9 ⋅ 10^−9^

**Table 12:** Comparison between entrainment parameter *f*(∞) obtained by numerical method and OHPM for *m* = 1 and different values of the parameters *S* and λ.

		entrainment parameter: *f*(∞)		
*S*	λ	*f*_numeric_(∞)	${\bar{f}}_{\text{OHPM}}\left(\infty \right)$	$\begin{array}{c} \text{relative error}=|f_{\text{numeric}}\left(\infty \right)-{\bar{f}}_{\text{OHPM}}\left(\infty \right)| \end{array}$
0.5	0.3	1.14796036697851	1.14796036700583	2.7 ⋅ 10^−11^
1	0.3	1.47146820418950	1.47146820422272	3.3 ⋅ 10^−11^
2	0.3	2.26317438627702	2.26317438629620	1.9 ⋅ 10^−11^
0.5	0.5	1.09219358570550	1.09219358570594	4.4 ⋅ 10^−13^
1	0.5	1.41421356237333	1.41421356238542	1.2 ⋅ 10^−11^
2	0.5	2.21431974337752	2.21431974339392	1.64 ⋅ 10^−11^
0.5	0.75	1.04010884613937	1.04010884613133	8.04 ⋅ 10^−12^
1	0.75	1.36285842819632	1.36285842818924	7.08 ⋅ 10^−12^
2	0.75	2.17476506480466	2.17476506481112	6.4 ⋅ 10^−12^

From Tables 2–12 we can summarize that the results obtained by means of OHPM are in very good agreement with the numerical results, but results obtained by means of HPM are relative good in comparison with the numerical results (see Table 8).

## Discussion

In order to analyze our results we illustrate the effects of slip parameter λ, and of suction parameters *S* for a fixed value of the nonlinear stretching parameter *m* = 1.

From the Table 11 we can deduce that the skin friction coefficient $-{\bar{f}}^{\prime \prime }\left(0\right)$ increases with *S*, whereas decreases with slip parameter λ. From the Table 12 it is clear that $\bar{f}$ at infinity increases with *S*, but decreases with λ.

**Fig 1:**
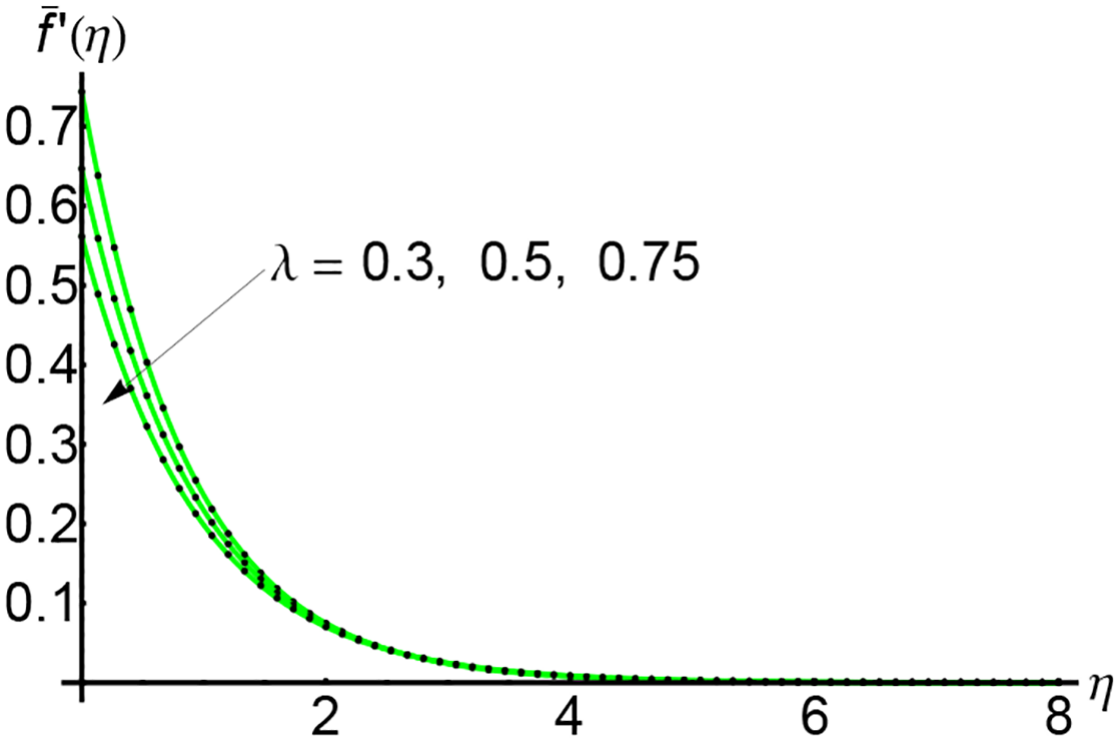
Variation of horizontal velocity ${\bar{f}}^{\prime }\left(\eta \right)$ with *η* for different values of slip parameter λ and for *S* = 0.5: —— numerical solution ………. OHPM solution.

**Fig 2:**
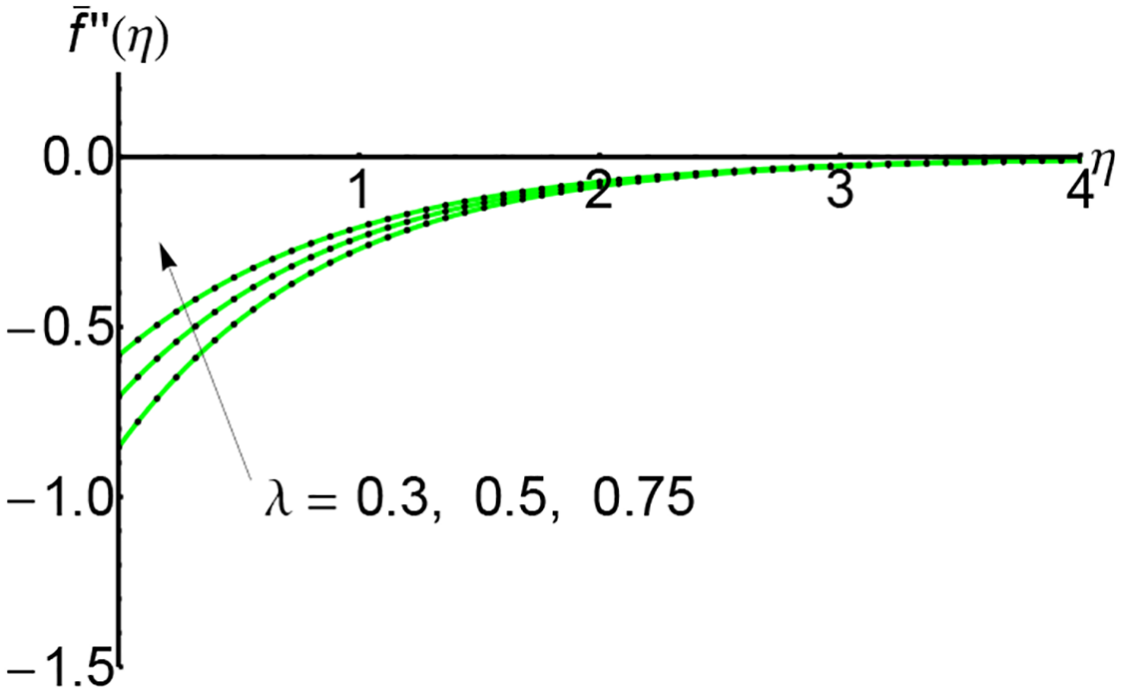
Variation of horizontal velocity ${\bar{f}}^{\prime }\left(\eta \right)$ with *η* for different values of slip parameter λ and for *S* = 1: —— numerical solution ………. OHPM solution.

**Fig 3:**
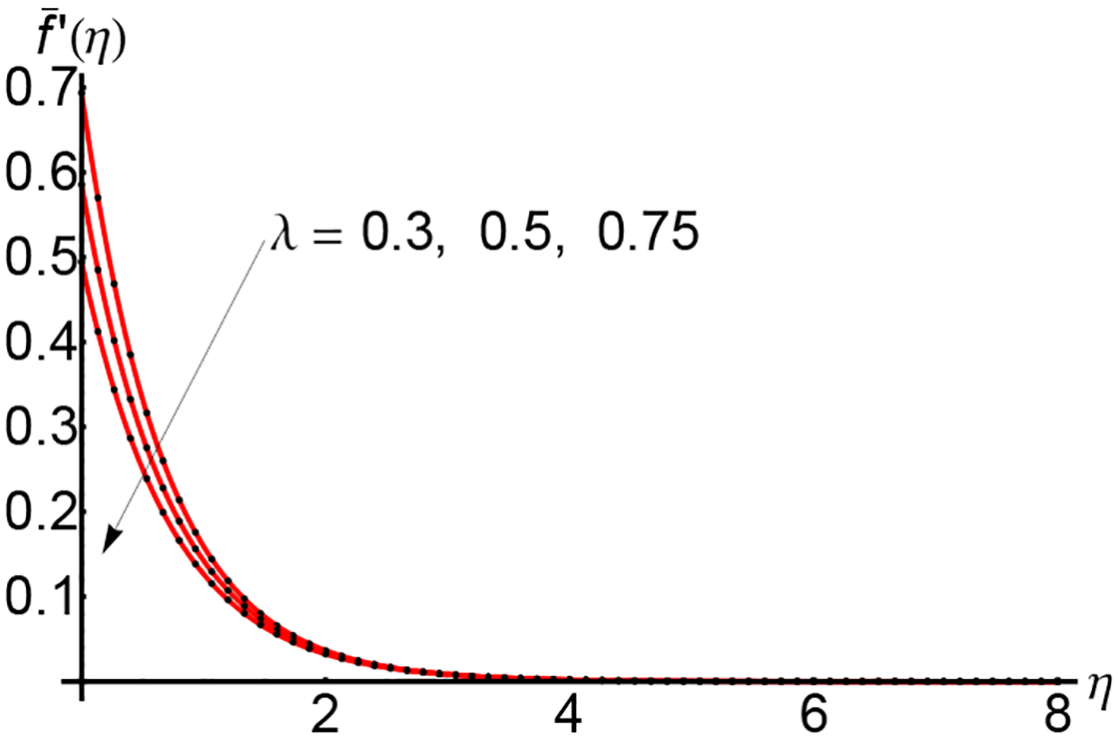
Variation of horizontal velocity ${\bar{f}}^{\prime }\left(\eta \right)$ with *η* for different values of slip parameter λ and for *S* = 2: —— numerical solution ………. OHPM solution.

In Figs 1, 2 and 3 are plotted the profiles of the horizontal velocity ${\bar{f}}^{\prime }\left(\eta \right)$ for *S* = 0.5, 1 and 2 respectively and for different values of the slip parameter λ. The horizontal velocity curves decrease with increasing of *η* and vanishes near by *η* = 5. With the increasing of slip parameter λ, the horizontal velocity decreases initially until *η* = 3 and then at a for distance from the wall it increases slightly.

**Fig 4:**
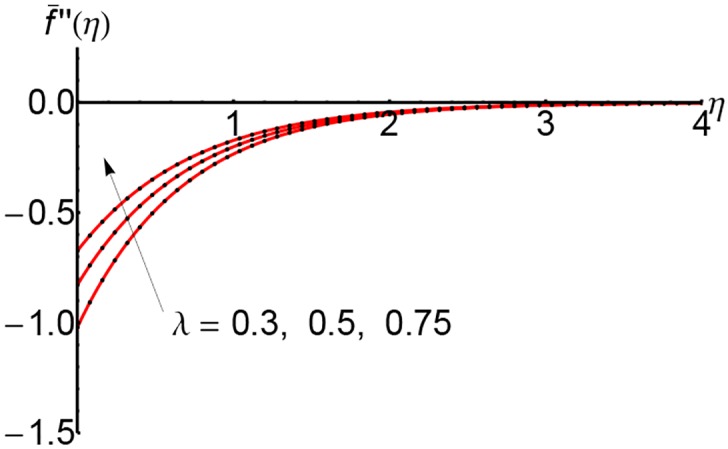
Variation of shear stress ${\bar{f}}^{\prime \prime }\left(\eta \right)$ with *η* for different values of slip parameter λ: —— numerical solution ………. OHPM solution.

**Fig 5:**
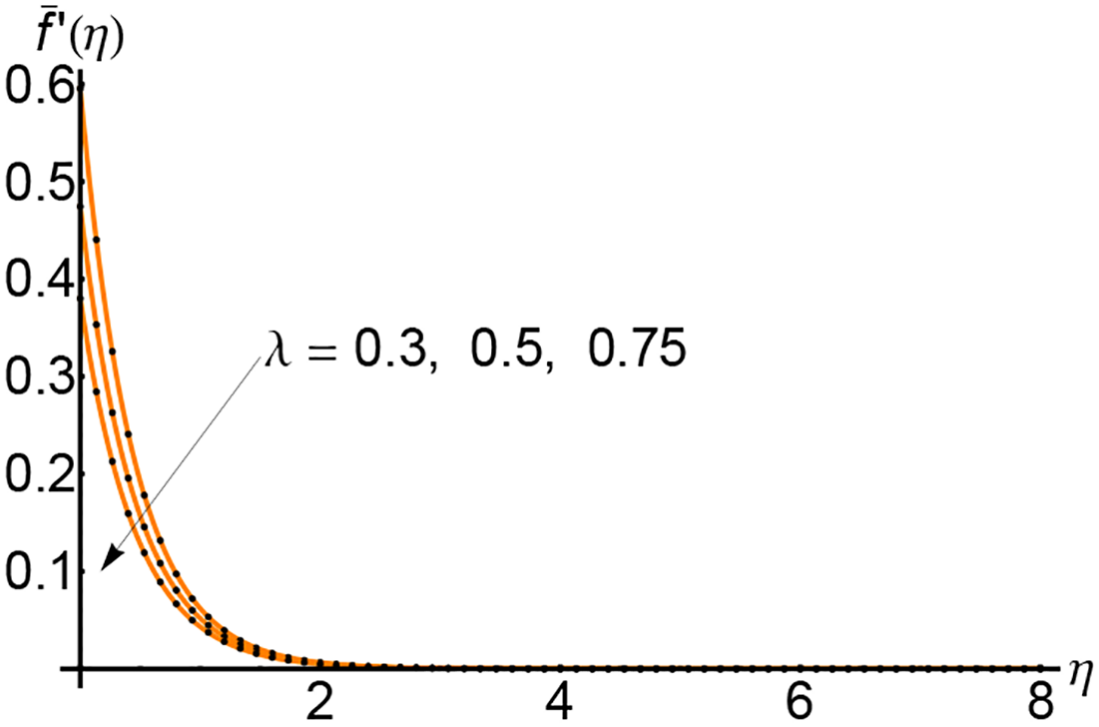
Variation of shear stress ${\bar{f}}^{\prime \prime }\left(\eta \right)$ with *η* for different values of slip parameter λ and for *S* = 1: —— numerical solution ………. OHPM solution.

**Fig 6:**
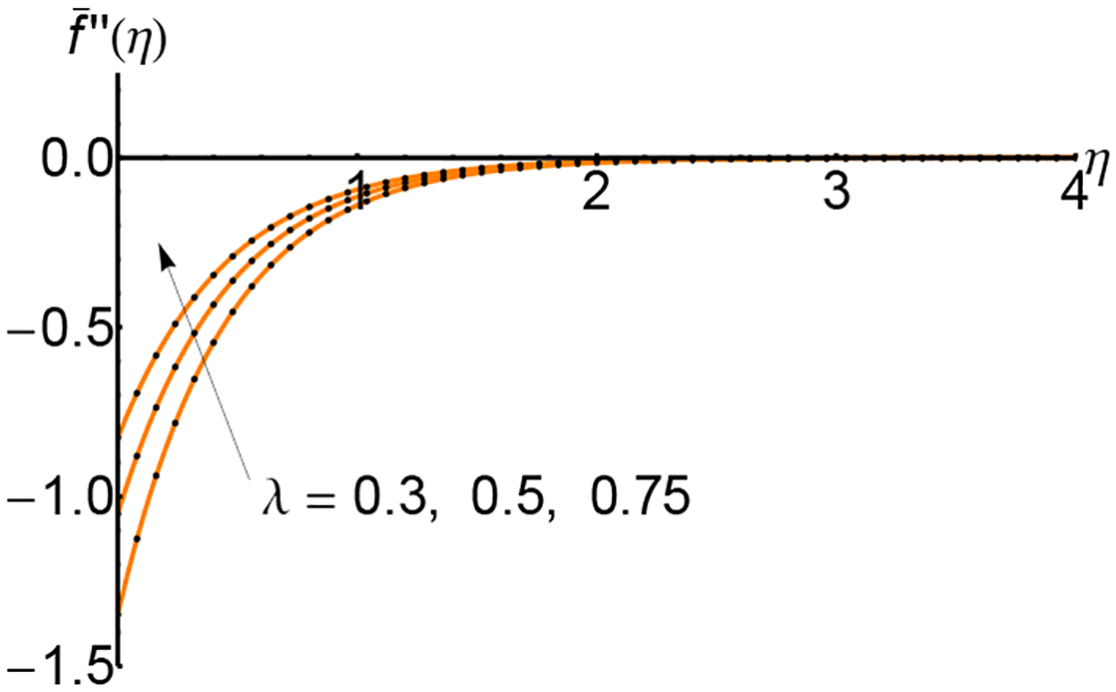
Variation of shear stress ${\bar{f}}^{\prime \prime }\left(\eta \right)$ with *η* for different values of slip parameter λ and for *S* = 2: —— numerical solution ………. OHPM solution.

Figs 4, 5 and 6 depict the shear stress ${\bar{f}}^{\prime \prime }\left(\eta \right)$ for *S* = 0.5, 1 and 2 respectively and for different values of the slip parameter λ. The shear stress increases with the increase of slip parameter. Also, the shear stress increases with an increase of the variable *η* and at a relative for distance from the wall it increases slightly.

**Fig 7:**
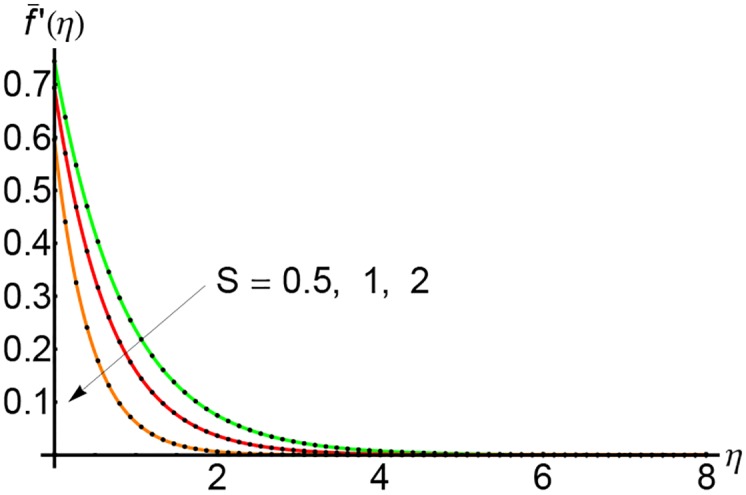
Variation of horizontal velocity ${\bar{f}}^{\prime }\left(\eta \right)$ with *η* for different values of suction parameter *S* and forλ = 0.3: —— numerical solution ………. OHPM solution.

**Fig 8:**
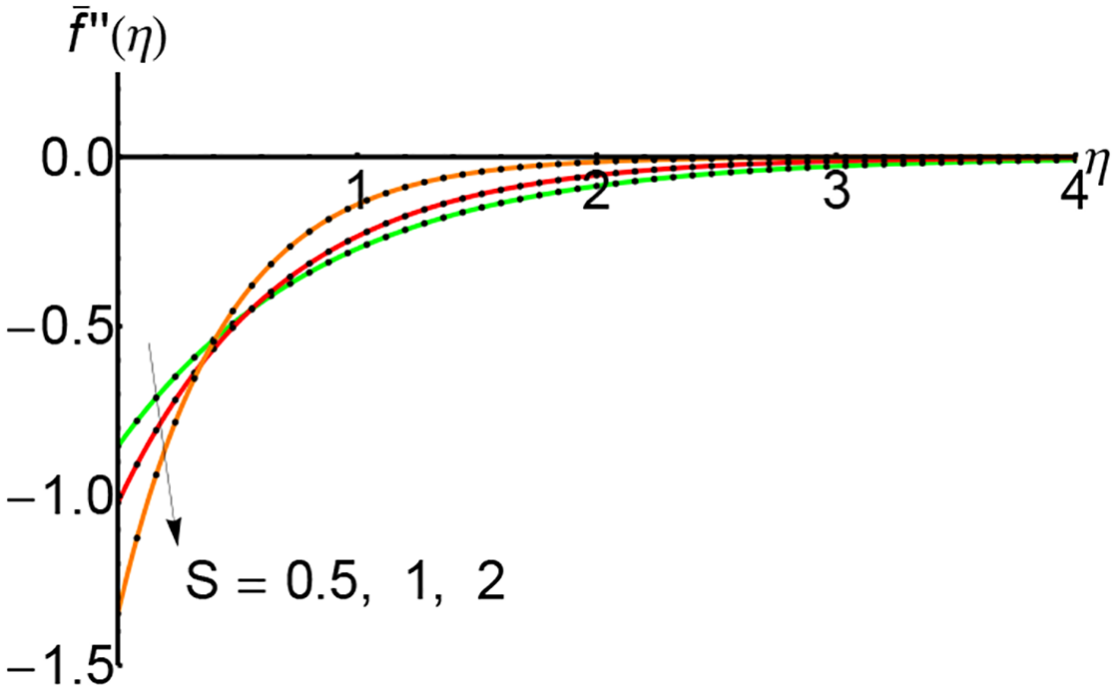
Variation of horizontal velocity ${\bar{f}}^{\prime }\left(\eta \right)$ with *η* for different values of suction parameter *S* and forλ = 0.5: —— numerical solution ………. OHPM solution.

**Fig 9:**
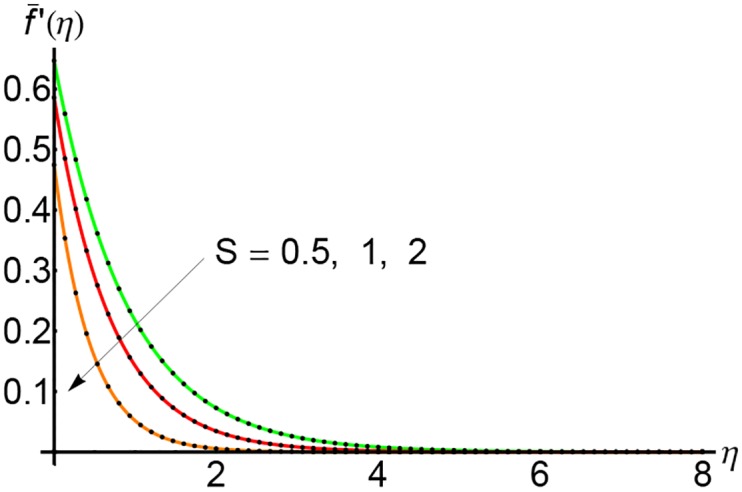
Variation of horizontal velocity ${\bar{f}}^{\prime }\left(\eta \right)$ with *η* for different values of suction parameter *S* and forλ = 0.75: —— numerical solution ………. OHPM solution.

Figs 7, 8 and 9 depict the effects of the suction parameter *S* on the velocity for different values of the slip parameter λ. In these cases, the horizontal velocity decreases with increasing of the suction parameter *S*. It is clear that the horizontal velocity decreases with increasing of the variable *η*.

**Fig 10:**
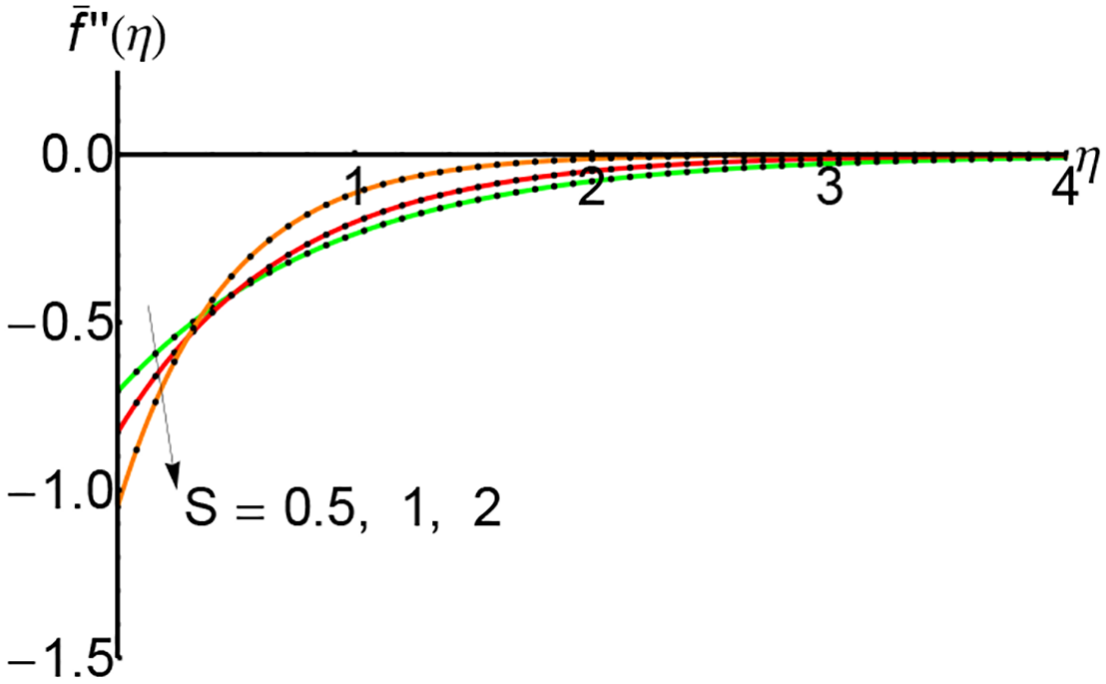
Variation of shear stress ${\bar{f}}^{\prime \prime }\left(\eta \right)$ with *η* for different values of suction parameter *S* and forλ = 0.3: —— numerical solution ………. OHPM solution.

**Fig 11:**
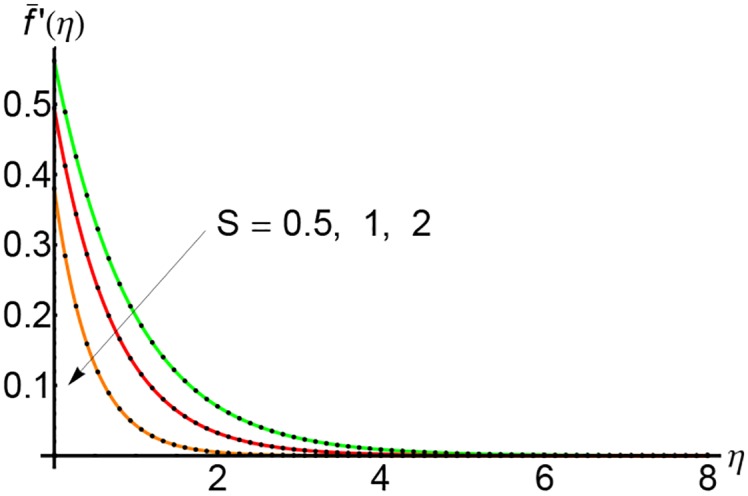
Variation of shear stress ${\bar{f}}^{\prime \prime }\left(\eta \right)$ with *η* for different values of suction parameter *S* and forλ = 0.5: —— numerical solution ………. OHPM solution.

**Fig 12:**
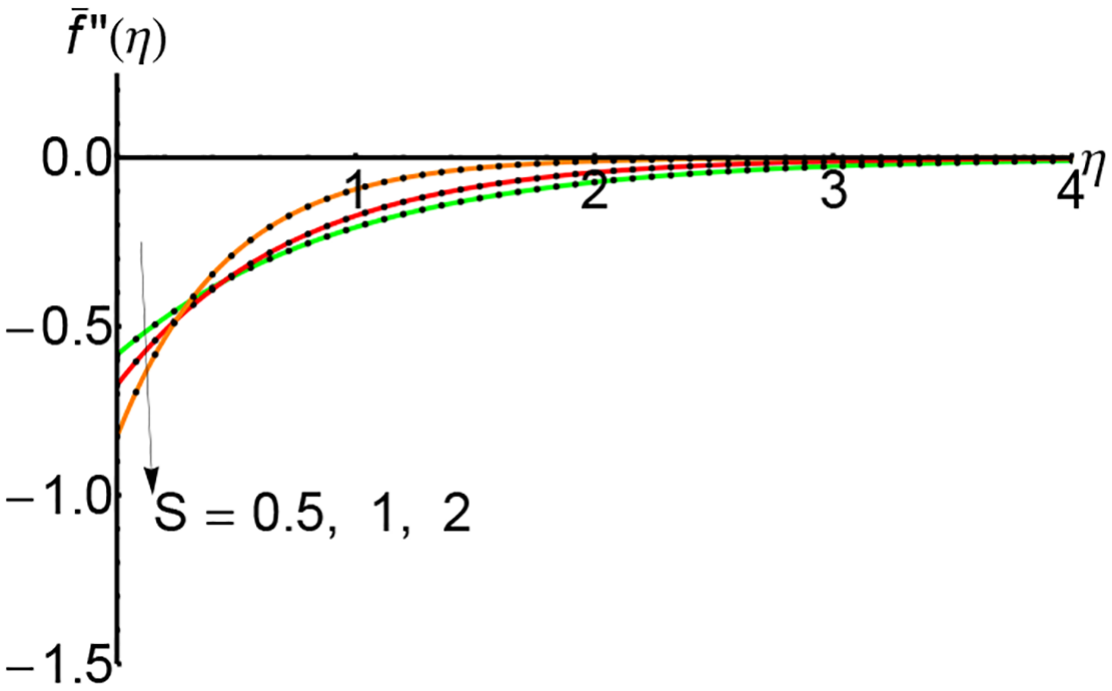
Variation of shear stress ${\bar{f}}^{\prime \prime }\left(\eta \right)$ with *η* for different values of suction parameter *S* and forλ = 0.75: —— numerical solution ………. OHPM solution.

Figs 10, 11 and 12 represent the shear stress profiles ${\bar{f}}^{\prime \prime }\left(\eta \right)$ and the effects of the suction parameter *S* for different values of the slip parameter. The shear stress curves decreases initially with to certain heights and then it increases slightly. The shear stress increases with an increase of the variable *η*.

**Fig 13:**
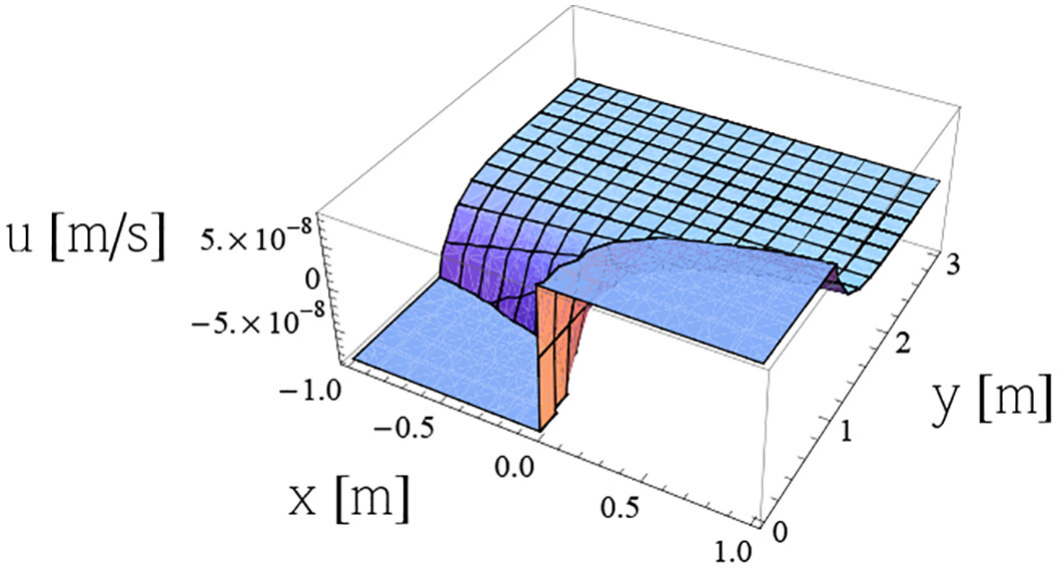
Variation of velocity component *u* from Eq (6) for *m* = 1, *S* = 0.5, λ = 0.3 for toluene at temperature 20°C.

**Fig 14:**
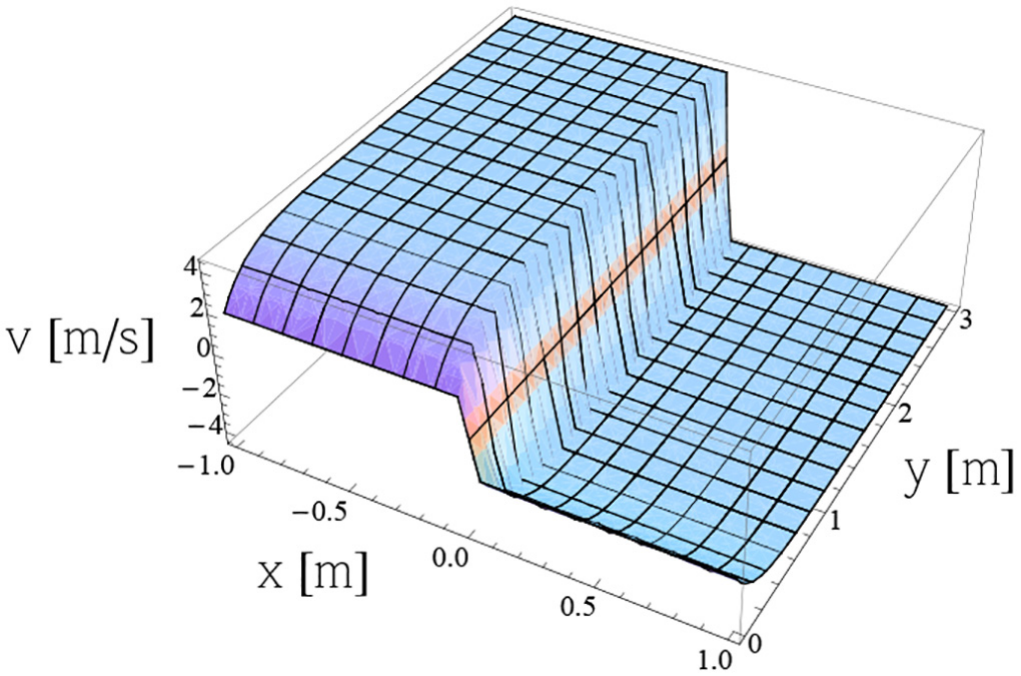
Variation of velocity component *v* from Eq (6) for *m* = 1, *S* = 0.5, λ = 0.3 for toluene at temperature 20°C.

Figs 13 and 14 represent the components *u* and *v* of the vector velocity respectively, obtained from Eq (6) for *m* = 1, *S* = 0.5, λ = 0.3, for toluene at temperature 20°C. (more details in S1–S14 Figs)

## Conclusions

In present work gives analytic approximate solutions for to the boundary layer flow equation over a stretching wall in presence of partial slip at the boundary. The effects of the slip parameter λ and suction parameter *S*, for fixed value of stretching parameter *m* are shown. In all cases, the velocity decreases and shear stress decreases with the variable *η*.

Optimal homotopy perturbation method (OHPM) is employed to find an analytical approximate solutions for this problem, but sometimes is used to find the exact solutions. The approximate solutions are compared with numerical solutions computed by means of the shooting method combined with the fourth-order Runge-Kutta method using Wolfram Mathematica 6.0 software. Our approach is valid even if the non-linear differential equation of the motion do not contain any small or large parameters. The propose procedure is based on a new construction of the homotopy and especially on the involvement of the convergence-control parameters via the auxiliary functions. These parameters lead to an excellent agrement of the approximate solutions with numerical results. On the other hand, we have freedom to choose the linear operator, auxiliary function and convergence-control parameters. Our technique is very effective, explicit, accurate and rapidly converging to the exact solution after only two iterations. Also, OHPM provides a simple but rigorous way to control and adjust the convergence of the approximate solution by means of some convergence-control parameters. It is worth mentioning that the proposed method is straightforward, concise and can be applied to other nonlinear problems.

## Supporting Information

S1 FigVariation of horizontal velocity ${\bar{f}}^{\prime }\left(\eta \right)$ with *η* for different values of slip parameter λ and for *S* = 0.5: —— numerical solution ………. OHPM solution.(PDF)

S2 FigVariation of horizontal velocity ${\bar{f}}^{\prime }\left(\eta \right)$ with *η* for different values of slip parameter λ and for *S* = 1: —— numerical solution ………. OHPM solution.(PDF)

S3 FigVariation of horizontal velocity ${\bar{f}}^{\prime }\left(\eta \right)$ with *η* for different values of slip parameter λ and for *S* = 2: —— numerical solution ………. OHPM solution.(PDF)

S4 FigVariation of shear stress ${\bar{f}}^{\prime \prime }\left(\eta \right)$ with *η* for different values of slip parameter λ and for *S* = 0.5: —— numerical solution ………. OHPM solution.(PDF)

S5 FigVariation of shear stress ${\bar{f}}^{\prime \prime }\left(\eta \right)$ with *η* for different values of slip parameter λ and for *S* = 1: —— numerical solution ………. OHPM solution.(PDF)

S6 FigVariation of shear stress ${\bar{f}}^{\prime \prime }\left(\eta \right)$ with *η* for different values of slip parameter λ and for *S* = 2: —— numerical solution ………. OHPM solution.(PDF)

S7 FigVariation of horizontal velocity ${\bar{f}}^{\prime }\left(\eta \right)$ with *η* for different values of suction parameter *S* and for λ = 0.3: —— numerical solution ………. OHPM solution.(PDF)

S8 FigVariation of horizontal velocity ${\bar{f}}^{\prime }\left(\eta \right)$ with *η* for different values of suction parameter *S* and for λ = 0.5: —— numerical solution ………. OHPM solution.(PDF)

S9 FigVariation of horizontal velocity ${\bar{f}}^{\prime }\left(\eta \right)$ with *η* for different values of suction parameter *S* and for λ = 0.75: —— numerical solution ………. OHPM solution.(PDF)

S10 FigVariation of shear stress ${\bar{f}}^{\prime \prime }\left(\eta \right)$ with *η* for different values of suction parameter *S* and for λ = 0.3: —— numerical solution ………. OHPM solution.(PDF)

S11 FigVariation of shear stress ${\bar{f}}^{\prime \prime }\left(\eta \right)$ with *η* for different values of suction parameter *S* and for λ = 0.5: —— numerical solution ………. OHPM solution.(PDF)

S12 FigVariation of shear stress ${\bar{f}}^{\prime \prime }\left(\eta \right)$ with *η* for different values of suction parameter *S* and for λ = 0.75: —— numerical solution ………. OHPM solution.(PDF)

S13 Fig Variation of velocity component *u* from Eq (6) for *m* = 1, *S* = 0.5, λ = 0.3 for toluene at temperature 20°C. (PDF)

S14 Fig Variation of velocity component *v* from Eq (6) for *m* = 1, *S* = 0.5, λ = 0.3 for toluene at temperature 20°C. (PDF)
