# Supplementary material for: Analytic Approximate Solutions to the Boundary Layer Flow Equation over a Stretching Wall with Partial Slip at the Boundary
Source: PLoS One. 2016 Mar 31;11(3):e0149334. doi: 10.1371/journal.pone.0149334 (PMC4816397; doi:10.1371/journal.pone.0149334)

Analytic approximate solutions to the boundary layer flow equation over a stretching wall with partial slip at the boundary

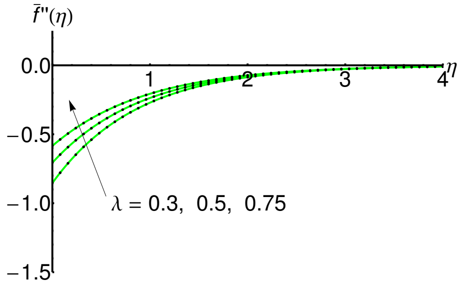

Supplement: S4 Fig — (PDF) [file pone.0149334.s004.pdf]
